# Long Shelf-Life Ready-to-Eat Plant-Based Whole Hard-Boiled Eggs: Low Allergenic and Regular Formulas

**DOI:** 10.3390/foods14132220

**Published:** 2025-06-24

**Authors:** Kanda Wongwailikhit, Suvimol Soithongsuk, Yupakanit Puangwerakul

**Affiliations:** 1 Department of Chemistry, Faculty of Science, Rangsit University, Pathum Thani 12000, Thailand; kanda.w@rsu.ac.th; 2 Innovative Research and Incubation of Entrepreneur Center, Rangsit University, Pathum Thani 12000, Thailand; suvimol.88@gmail.com; 3 Faculty of Food Technology, College of Agricultural Innovation and Food Technology, Rangsit University, Pathum Thani 12000, Thailand

**Keywords:** mung bean protein, wheat protein, vegan nutrition, thermal processing, gamma irradiation, shelf-stable foods, protein alternative

## Abstract

This study aimed to develop a shelf-stable, plant-based whole hard-boiled egg analogue, available in both regular and low-allergenic versions. Six plant proteins—soy, mung bean, pea, rice, potato, and wheat—were formulated into egg white and yolk components, with mung bean and wheat proteins showing the most promising sensory and visual qualities. Two preservation methods, thermal pasteurization (75–85 °C, 15–20 min) and gamma irradiation (2–5 kGy), were applied to extend shelf life while maintaining product quality. Thermal treatment at 75 °C for 15 min and gamma irradiation at 3.5 kGy were identified as optimal conditions, balancing sensory acceptability and microbial safety. Sensory evaluation by 100 untrained panelists revealed favorable scores for appearance, texture, and overall liking, without significant differences among selected formulations (*p* > 0.05). Accelerated shelf life testing and Q10 modeling predicted a shelf life of 188 days for thermally pasteurized eggs and 253 days (8.42 months) for gamma-irradiated eggs at 30 °C. These results demonstrate the feasibility of developing a consumer-acceptable, plant-based hard-boiled egg analogue with extended ambient shelf life.

## 1. Introduction

Rising global interest in plant-based foods, especially among vegans, health-conscious consumers, and those with egg allergies, is catalyzing innovation in convenient and nutritionally adequate egg alternatives [1,2,3,4]. Although powdered vegan eggs exist, their limited preparation flexibility and need for additional cooking reduce their appeal for consumers seeking convenience [5]. In response, this study developed long-shelf-life, ready-to-eat (RTE) plant-based hard-boiled eggs with an appearance and shape closely resembling real eggs. Six alternative proteins, such as mung bean, pea, hemp, soy, and wheat, were tested. Both low-allergenic and regular formulations were selected to meet the needs of both consumer groups.

To address this evolving demand, the current study was designed with two primary goals: (1) to develop a whole plant-based egg analogue with a long shelf life, and (2) to provide consumers with options for both regular formula and a low allergenic formula for reducing the presence of major allergens listed by FAO/WHO.

These products typically use proteins from pulses such as soy, pea, lentil, lupine, and chickpea, which provide functionalities like gelling, emulsification, and foaming, and are available in forms like flour and protein isolates [6]. Among these, soy is the most commonly used due to its high nutritional quality, as seen in products like tofu and texturized vegetable protein [7]. Additionally, wheat gluten provides a meat-like texture, while mushrooms add chewiness. Pea protein and oilseed proteins from rapeseed and canola also help create meat-like textures [3].

There are several important developments in RTE plant-based eggs worth mentioning. Lu, Lee, and Yang (2022) created a plant-based egg analogue using chickpea flour, soy isolate, and κ-carrageenan, which enhanced gel strength and reduced viscosity to better mimic omelets [8]. In 2023, they further optimized these analogues by adjusting hydroxypropyl methylcellulose (HPMC) levels and starch-protein ratios, achieving textures closely resembling traditional eggs, particularly with soy protein isolate (SPI) and potato starch (PS) at an 8:6 ratio [9]. Xiangfang Hu and Zong Meng (2024) introduced a flourless alternative using protein and curdlan, with higher protein ratios (above 8:2) closely mimicking real egg thermal properties in applications like steamed egg custards and omelets [10]. These products are designed to mimic the texture and appearance of traditional hard-boiled eggs, featuring a distinct white part and a yolk-like center [11] and provide the expected shelf life under refrigeration for 90–120 days [12].

Our prior work [13] focused on rice-protein-based vegan egg powders. The current study significantly extends that work by developing whole hard-boiled egg analogues using alternative proteins (e.g., mung bean and wheat), optimizing both low-allergenic and regular formulations, and introducing shelf-life prediction through thermal and gamma sterilization technologies.

With a shelf life of up to 16 months at ambient temperature, the product presents a viable alternative among plant-based protein sources. It demonstrates strong commercial potential in terms of color, texture, and sensory attributes, as well as a high plant-based protein content. However, differences in composition and bioavailability compared to real eggs remain to be fully evaluated. The product also exhibits higher levels of beneficial compounds such as polyphenols, antioxidants, and bioactive peptides compared to both real eggs and commercial vegan eggs. Furthermore, we have successfully developed a whole plant-based boiled egg using rice protein as the base. This product closely resembles a real egg, featuring a white egg surrounding a centered yellow yolk, addressing the growing consumer demand for plant-based alternatives that closely mimic traditional animal products [14].

Although rice protein is widely considered hypoallergenic, it has been reported as a rare allergen in pediatric populations [15]. Therefore, this study also explores alternative proteins to offer broader formulation options for sensitive consumers. Although several recent studies have explored plant-based egg alternatives, most focus on liquid or scrambled analogues. In contrast, this study presents a novel formulation of a ready-to-eat, shelf-stable whole hard-boiled egg analogue with separate yolk and white components. Furthermore, we investigate both conventional (thermal) and advanced preservation methods (gamma irradiation), allowing for long shelf-life at ambient conditions. Importantly, the study includes allergen-reduced formulations using mung bean and hemp proteins and provides comprehensive nutritional, physical, and sensory analyses. These features collectively distinguish our work from previous research in the field. We utilize various alternative protein sources to provide consumers with a variety of choices while maintaining nutritional value, affordability, and sustainability, thereby enhancing consumer acceptance [16]. The proposed protein sources include low allergenic options such as mung bean [17,18] and hemp [19], as well as allergenic proteins suitable for regular formulations, such as soy [20], pea [21], and wheat [22]. The study is designed to test different ratios of alternative proteins to rice protein, building on the successful rice protein formula. Additionally, extending the shelf life of plant-based vegan eggs, which often spoil more quickly than traditional eggs, is crucial to meet future demand and drive market growth.

This work aims to develop RTE plant-based whole hard-boiled eggs derived from locally sourced natural proteins, including mung bean and hemp (for low allergenic options), and soy, pea, and wheat (for regular options). The product formulation aims to offer a convenient, nutritious, and long-lasting alternative to traditional eggs while achieving an acceptable level of consumer satisfaction.

## 2. Materials and Methods

### 2.1. Ingredients and Formulations for the Plant-Based Egg

Rice protein hydrolysate was obtained from the Innovative Research and Incubation of Entrepreneur Center, Faculty of Food Technology, Rangsit University, Pathum Thani, Thailand. Mung bean protein, pea protein, soy protein, wheat protein, and hemp protein were purchased from Fuzhou Xinchuang Bio-Technology Co., Ltd., Fuzhou, China. Commercial gellan gum and nisin were purchased from Krungthep Chemical Company Limited, Bangkok, Thailand. Mashed pumpkin was prepared from fresh pumpkin purchased from local grocery outlets, steamed, and mashed in the laboratory. Gamma irradiation was performed at the Thailand Institute of Nuclear Technology (Public Organization), Pathum Thani, Klong 5, Thailand. All ingredients were food-grade.

This study systematically developed and evaluated the shelf life of ready-to-eat (RTE) plant-based whole hard-boiled eggs. The basic formulations for the plant-based egg white and yolk were adapted from a previously published study by Puangwerakul et al. (2024) [14], with brief details provided below.

The plant-based egg white mixture was prepared using the following ingredients: 35% rice protein hydrolysate and 4% rice protein isolate, which served as the primary protein base; 34% rice malt flour, functioning as both a carbohydrate source and texture enhancer; and 17% PEN EGG—a commercial vegan egg powder comprising 55% mung bean, 15% rice protein, 10% broken Thai rice malt, 7% oat bran, 6% garlic powder, 3% yeast extract, 2% salt, 1.5% pepper, and gellan gum. PEN EGG was incorporated to enhance emulsification, improve binding, and mimic the functionality of traditional egg components. The formulation also included 5% dried bio-cellulose powder to support gel structure and moisture retention, 3% dried yeast for flavor and nutritional enrichment, 1.2% angkak red rice malt powder as a natural colorant, 0.2% salt for seasoning, and 0.6% gellan gum as a gelling agent. Nisin was added at a concentration of 25 mg/kg as a natural preservative. The ingredients were mixed in a high-speed blender (Toshiba Real Smooth Series BL-T70PR2, Tokyo, Japan) using medium-high speed for 5 min. The pH of the blended egg white mixture was 6.42 ± 0.1, and water activity (a_w_) was 0.79 ± 0.02, measured using a portable meter (AquaLab 3TE, Meter Group, Pullman, WA, USA). These values were recorded prior to steaming. All components were processed under standardized environmental conditions (25 ± 2 °C ambient temperature).

For the yolk component, the same base formulation was blended with mashed pumpkin in a 1:1 weight ratio (*w*/*w*). The addition of mashed pumpkin contributed both the characteristic orange-yellow coloration and the creamy texture typical of egg yolk. The egg yolk mixture, after the addition of mashed pumpkin, had a pH of 6.3 ± 0.1 and a_w_ of 0.79 ± 0.03. The yolk mixture was filled into spherical molds and steamed at 100 °C for 15 min, or until a firm gel consistency was achieved.

To assemble the plant-based whole hard-boiled egg, half of the egg white mixture was poured into an egg-shaped silicone mold. A cavity was created at the center to insert the pre-steamed yolk. The remaining egg white mixture was then added to fully enclose the yolk, completing the egg structure. The assembled eggs were processed under controlled environmental conditions (25 ± 2 °C ambient temperature) and steamed for an additional 15 min to ensure complete integration and uniform texture.

Finally, the cooked plant-based eggs were vacuum-sealed in retort pouches and sterilized at 120 °C, achieving a sterilization value (Fo) of 6 min, followed by natural cooling to below 35 °C.

### 2.2. Development of Low Allergenic and Regular Formulations

Two formulations of plant-based eggs were developed: (1) Low allergenic and (2) Regular.

For the low allergenic formula, mung bean and hemp proteins were tested, while soy, pea, and wheat proteins were used for the regular formula. Various ratios of these proteins to rice protein were tested. Quality analyses for the physical characteristics included texture, color. Texture firmness was measured using a penetrometer as described in previous studies [23]. Color measurement was conducted using a chromameter (Minolta CR-10, Tokyo, Japan) to determine the L* (lightness), a* (redness), and b* (yellowness) values of the products [24].

### 2.3. Study of Preservation Methods for Shelf-Life Extension

The shelf-life extension study compared two preservation methods: thermal processing and gamma irradiation. Thermal processing was conducted at 75 °C, 80 °C, and 85 °C for durations of 15 and 20 min [25]. Gamma irradiation was performed at the Thailand Institute of Nuclear Technology using a multipurpose Co-60 irradiator, Paul Stephens Consultancy Ltd. of Bristol, UK [26]. Samples in sealed plastic bags were exposed to doses of 0, 2, 3.5, and 5 kGy at room temperature, with an average dose rate of 2.6 Gy/hr. Quality analyses, including texture, color, sensory evaluation, and microbiological assessment, were conducted to ensure the preservation of product quality.

### 2.4. Shelf Life Determination

Shelf life determination is for assessing and identifying the storage time for a food product to remain safe and retain its desired sensory, chemical, and microbial characteristics. The samples were subjected to accelerated shelf life testing (ASLT) at elevated temperatures for four months [27]. Weekly analyses assessed physical characteristics, color, texture, microbial quality, chemical quality, and sensory evaluation. It is noted here that samples were prepared with 0.1% acetic acid as a natural preservative before testing at temperatures of 45 °C and 55 °C for thermal sterilization, and 40 °C and 50 °C for gamma-ray sterilization.

### 2.5. Microbial Analysis

Microbial analysis was conducted to assess the total microbial count of the plant-based hard-boiled eggs. The microbial analysis included enumerating total aerobic bacteria, yeast, and mold, and specific pathogens such as *E. coli*, *Clostridium perfringens*, *Staphylococcus aureus*, *Salmonella* spp., and *Bacillus cereus*. All microbial counts were performed in triplicate. Results were expressed in colony-forming units per gram (CFU/g) and compared against acceptable limits to ensure food safety. The Standard Plate Count method was used, with samples incubated at 35 °C and 55 °C [28].

### 2.6. Lipid Oxidation Test

Lipid oxidation was assessed by measuring 2-thiobarbituric acid (TBA) levels [29]. Egg samples were homogenized and reacted with TBA reagent to form a colored complex with malondialdehyde (MDA). Measurements were conducted in triplicate. The MDA content was quantified using spectrophotometric analysis to evaluate the degree of lipid oxidation.

### 2.7. Firmness Measurement

Firmness was measured using a penetrometer (Humboldt MFG.CO A8TM, Elgin, IL, USA) by recording the depth (in mm) of probe penetration under a defined force (500 g) over 5 s. Each sample was measured in triplicate.

### 2.8. Sensory Evaluation and Consumer Acceptance Testing

A consumer acceptance test was conducted with 100 untrained panelists: 50 general consumers (20 male, 30 female) and 50 self-identified vegans (24 male, 26 female), resulting in a panel of 44 males and 56 females (mean age = 35.1 ± 13.9). Participants were recruited via local advertisement and screened for regular egg consumption (general group) or animal product avoidance (vegan group). All panelists provided informed consent, and the study was approved by the university ethics board (COA No. RSUERB2022-067). The test used a 9-point hedonic scale (1 = dislike extremely, 9 = like extremely) to assess the following attributes: appearance, color, smell, taste, texture, and overall liking [30].

All samples were coded with three-digit blinding codes and served in randomized order. Evaluation was conducted in individual sensory booths under neutral white lighting, at 25 ± 2 °C. Samples were served at room temperature, and participants were provided with water. One testing session was conducted for each participant, in which all six sample types were evaluated within approximately 20 min.

### 2.9. Statistical Analysis

All experiments were performed in triplicate unless otherwise stated. Data were analyzed using SPSS software version 25.0. One-way ANOVA was conducted, followed by Tukey’s Honestly Significant Difference (HSD) test to identify significant differences between means. Statistical significance was set at *p* < 0.05.

## 3. Results

**Figure 1:**
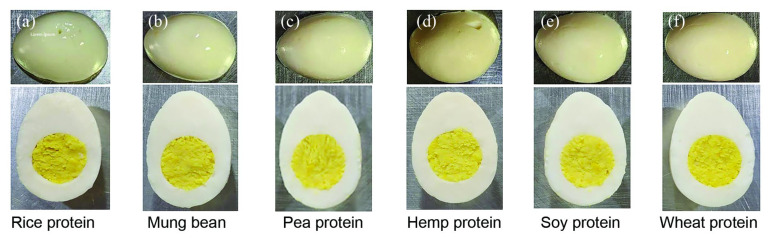
Visual comparison of whole and halved plant-based hard-boiled eggs prepared using different protein sources ((**a**–**f**): rice, mung bean, pea, hemp, soy, wheat). Note: Samples ((**a**): rice, (**d**): hemp) exhibit slight surface irregularities due to prototype molding limitations. These do not reflect the sensory or textural outcomes as measured in the main analysis.

Figure 1 presents visual comparisons of whole and halved plant-based hard-boiled eggs formulated with various proteins, highlighting surface sheen and color uniformity relevant to consumer appeal, both whole (top row) and halved (bottom row), prepared using a base formula combined with various plant proteins. Images demonstrate surface sheen and color variation, particularly the shinier appearance, which correlates with observed L values in Table 1. The products include (a) rice protein, (b) mung bean protein, (c) pea protein, (d) hemp protein, (e) soy protein, and (f) wheat protein. The mung bean-based formulation exhibited the highest surface gloss, consistent with elevated L* values, indicating superior visual appeal, with the rice protein product (Figure 1a) also showing some shine, though less pronounced. The products made with pea, hemp, soy, and wheat proteins exhibit similar appearances, lacking the shine seen in the mung bean and rice protein products. These visual results are one of the key factors influencing the product and will be considered alongside the instrumental color measurements presented in Table 1 in the following section.

### 3.1. Physical and Color Properties of Plant-Based Whole Hard-Boiled Eggs Formulated with Different Proteins

The physical and color properties of plant-based whole hard-boiled eggs formulated with different proteins were assessed. Instrumental color measurements from Table 1 revealed notable differences among formulations in terms of lightness (L*), redness (a*), and yellowness (b*). The soy protein formulation exhibited the highest L* value (82.1 ± 1.5), indicating the brightest surface, followed closely by mung bean (75.7 ± 0.3) and rice protein (73.0 ± 0.5). These values suggest a relatively light and visually appealing appearance. In contrast, the hemp-based sample recorded the lowest L* value (68.2 ± 1.5), corresponding to a darker overall tone.

Regarding b* values, which reflect yellowness and contribute to yolk realism, the wheat protein formulation achieved the highest score (29.2 ± 1.0), enhancing its visual resemblance to cooked egg yolks. Hemp protein again recorded the lowest b* value (22.4 ± 2.2), resulting in a less vibrant appearance. Differences in a* (redness) were minimal and not statistically significant, contributing little to the overall color profile.

These instrumental findings correspond closely with the visual outcomes in Figure 1. The mung bean (Figure 1b) and rice protein (Figure 1a) formulations displayed greater surface gloss and lighter coloration, visually consistent with their higher L* values. In contrast, hemp and pea protein samples appeared dull and matte, reflecting their lower L* and b* values. Although soy protein showed the highest L* value numerically, its dull surface finish reduced its perceived brightness in comparison to mung bean and rice samples.

The observed differences in color and gloss can be attributed to the distinct molecular and functional characteristics of the proteins used. Mung bean and wheat proteins tend to form more cohesive gels upon heating, promoting smooth, glossy surfaces and consistent pigment distribution. In contrast, hemp protein appears to form a looser matrix with lower structural integrity, which may contribute to uneven surface reflection and muted coloration. Additionally, color differences, especially in b* values, may arise from inherent pigment composition in the raw ingredients and from thermal interactions during sterilization, such as Maillard browning reactions.

### 3.2. Sensory Test Results

**Figure 2:**
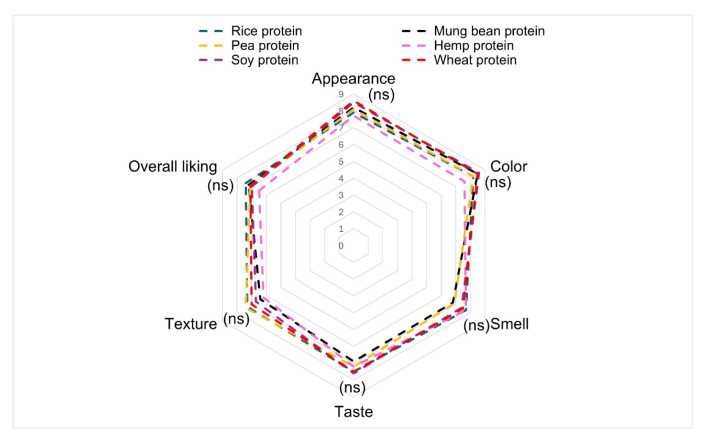
Radar plot of sensory attributes (mean values only) for six plant-based egg formulations. Standard deviations are provided in Table 1.

Sensory evaluation results (Table 2 and Figure 2) provide insight into consumer perception of plant-based whole hard-boiled eggs formulated with different protein sources using a 9-point hedonic scale. Flavor-related attributes (smell, taste, and texture) showed more variation, although no statistically significant differences were observed across samples. The appearance scores were consistently high, ranging from 7.7 to 8.6, within the acceptable range (>6.0), with no statistically significant differences (*p* > 0.05), indicating that all products were visually acceptable. Notably, the wheat and soy protein samples received the highest mean appearance ratings (8.6 ± 0.4 and 8.5 ± 0.2, respectively), followed closely by mung bean (8.2 ± 0.4), consistent with their instrumental L* and b* values, which reflect lightness and yellowness, respectively. Wheat proteins generally performed well across all categories, while mung bean formulations received slightly lower texture scores (6.4 ± 0.5) with statistically significant differences (*p* < 0.05).

Sensory scores also varied due to flavor contributions such as soy and hemp proteins may introduce beany or grassy notes, which impact taste and aroma ratings. These compositional differences collectively influenced consumer acceptance and overall liking. Although sensory differences between formulations were not statistically significant (*p* > 0.05), the practical difference between a score of 6.1 (barely acceptable) and 7.5 (strongly liked) may be commercially meaningful, especially in competitive retail environments. Thus, future studies should include consumer purchase intention testing and larger demographic diversity to validate product-market fit.

### 3.3. Protein and Oil Contents

The total protein and oil content (AOAC, 2000 method) of the selected formulas for plant-based eggs are illustrated in Table 3.

Regarding the required total protein and oil in g/egg (nutritional values of protein 6–7 g and oil < 1.5 g/egg), it was found that all formulas meet the required protein content. The mung bean and wheat protein-based eggs slightly exceed the upper limit, with 7.08 ± 0.94 g and 7.08 ± 1.24 g of protein, respectively, but remain within an acceptable range considering the standard deviation.

Regarding oil content limitations, the mung bean and wheat protein-based eggs exhibited acceptable oil levels (<1.5 g/egg), with 1.29 ± 0.35 g and 0.72 ± 0.10 g of oil, respectively. Meanwhile, the rice and pea protein-based eggs, although high in protein (7.38 ± 1.34 g and 7.23 ± 1.15 g), have oil contents that exceed the acceptable limit, with values of 1.92 ± 0.44 g and 1.98 ± 0.36 g, respectively. The soy protein-based egg meets the oil content requirement with 0.36 ± 0.11 g, and the hemp protein-based egg, despite meeting the nutritional criteria with 6.77 ± 0.93 g of protein and 0.36 ± 0.13 g of oil, was found to have poor firmness, making it less desirable.

Therefore, based on the criteria of total protein and oil content, among those alternative plants, mung bean and wheat proteins are the most suitable choices for preparing plant-based whole hard-boiled eggs under the specified nutritional restrictions.

### 3.4. Preservation of Plant-Based Whole Hard-Boiled Eggs

To ensure product stability and safety over extended storage, two preservation methods, thermal pasteurization and gamma irradiation, were systematically evaluated. The following sections detail the effects of each method on the physical, sensory, and microbial properties of the plant-based whole hard-boiled eggs.

#### 3.4.1. Thermal Preservation: Optimization of Pasteurization Conditions

The experiments aimed to determine the optimal pasteurization conditions for the Preservation of both low-allergenic and regular plant-based whole hard-boiled eggs made with mung bean and wheat proteins, respectively. Physical, sensory, and microbiological qualities were assessed post-heating to identify the most effective conditions for maintaining product quality while ensuring microbial safety [31].

For the low-allergenic plant-based eggs made with mung bean protein, the results in Table 4 show that the designed heat levels effectively eliminated all target microorganisms. Heat treatments maintained acceptable physical quality, although higher temperatures and longer durations led to perceptible texture softening and aligned with the acceptable criteria in the last column. The optimal condition selected was 75 °C for 15 min, as it used the least energy and the shortest time while maintaining acceptable sensory qualities and meeting safety standards. Higher temperatures and longer durations led to perceptible texture softening and reduced sensory scores, as shown in Table 4, making 75 °C for 15 min the most suitable for preserving both quality and safety. Similarly, for the regular plant-based eggs made with wheat protein, all heat treatments effectively eliminated target microorganisms (Table 5). However, increased temperature and duration resulted in reduced firmness and noticeable texture softening, as shown by sensory and instrumental measurements. The optimal condition for both low allergenic and regular formulations was 75 °C for 15 min, preserving desirable texture and sensory attributes while ensuring microbial safety. All tested conditions met the criteria for acceptable sensory and physical quality, with no statistically significant differences in most sensory attributes (*p* ≤ 0.05), except for texture at higher temperatures.

These findings support the use of 75 °C for 15 min as the optimal pasteurization condition for extending the shelf life of plant-based whole hard-boiled eggs while maintaining their quality and safety.

#### 3.4.2. Gamma Irradiation Preservation: Optimization of Sterilization Conditions

For both low-allergenic mung bean protein and regular wheat protein plant-based whole hard-boiled eggs, the designed gamma radiation levels did not significantly affect the physical quality (see Table 6 and Table 7). However, sensory attributes were negatively impacted, with scores decreasing as radiation doses increased. Despite this, consumers still accepted the 2 and 3.5 kGy levels, as indicated by sensory scores remaining above the acceptable threshold (>6 on a 9-point scale).

As shown in Table 6, the firmness of mung bean protein eggs remained relatively stable across different radiation levels, with values ranging from 142.0 ± 2.0 mm to 145.0 ± 1.5 mm. Color parameters (L*, a*, b*) also showed minimal variation. Sensory attributes such as appearance, color, smell, taste, texture, and overall liking decreased with higher radiation doses, but the 2 and 3.5 kGy levels still met the acceptable range. The 3.5 kGy level was particularly effective in microbial sterilization, completely destroying all target microorganisms, making it the optimal preservation method.

Table 6 shows that the firmness of wheat protein eggs also remained stable, with values ranging from 126.8 ± 0.8 mm to 128.3 ± 1.1 mm. Color parameters (L*, a*, b*) exhibited minor changes. Similar to mung bean protein, sensory attributes for wheat protein eggs declined with increasing radiation doses, but the 2 and 3.5 kGy levels were still within the acceptable range. The 3.5 kGy level effectively sterilized all microbial groups, confirming its suitability as the best preservation method for plant-based eggs. In conclusion, the 3.5 kGy level was the most effective in ensuring microbial safety, making it the preferred method for preserving both low allergenic mung bean protein and regular wheat protein plant-based whole hard-boiled eggs.

### 3.5. The Shelf-Life Estimation

The shelf-life estimation in this study was based on the Q_10_ model, commonly applied in thermally processed or irradiated food systems to project deterioration rates as a function of storage temperature [27]. The formula used is:

$$\mathrm{Shelf}\;\mathrm{life}\;\mathrm{at}\;T_{2}=\mathrm{Shelf}\;\mathrm{life}\;\mathrm{at}\;T_{1}\times \;Q_{10}^{\frac{\left(T_{1-}T_{2}\right)}{10}}$$

where T_1_ is the accelerated test temperature, and T_2_ is the storage temperature. We adopted a Q_10_ value of 2.0, which is typical for lipid oxidation and microbial degradation in food matrices under non-enzymatic degradation processes such as texture and flavor deterioration in low-moisture or sterilized products [28]. Calculations were based on degradation endpoints defined by TBA levels, texture changes, and microbial thresholds.

#### 3.5.1. Shelf Life Estimation: Thermal Pasteurization:

**Figure 3:**
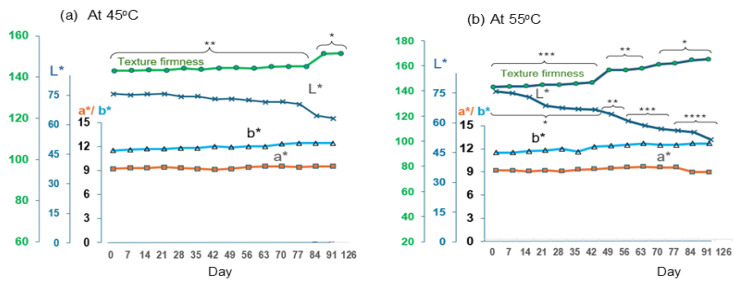
The quality changes for thermal pasteurized plant-based whole hard-boiled egg products: (**a**) at 45 °C and (**b**) at 55 °C. Groups of multiple data points are shown with horizontal braces to indicate significant differences between groups at *p* < 0.05 (*, **, ***, ****). Data are provided in Supplementary Materials Table S1.

The results for shelf life determination for both the product preserved under thermal pasteurization and gamma radiation were monitored periodically. The various analyses, namely microbiological (CFU/g), color (L*, a*, b*), texture firmness, and TBA, are shown in combination curves for different axes. Figure 3 is for the products preserved by thermal processing and tested at temperatures of 45 °C and 55 °C. Note that the data are uploaded as Supplementary Materials. The data derived from these experiments were then used to predict shelf life at lower, more practical storage temperatures, leveraging the Q_10_ model for estimating deterioration rates.

As shown in Figure 3a,b, there is no observed difference in a* (redness) and b* (yellowness) over the test period for both temperatures. However, marked changes in L* (lightness) were evident, with shelf-life endpoints reached by day 84 at 45 °C and day 49 at 55 °C, as indicated by texture degradation and microbial growth (Table 8). These findings suggest a moderate risk of spoilage under elevated temperature conditions. TBA levels remained below the threshold of 50 mmole/kg (indicating the onset of rancid odors) until days 84 at 45 °C and 49 at 55 °C, which suggests acceptable oxidative stability up to these points.

The application of Q_10_ modeling, calculation steps are detailed in Supplementary Materials (Supplementary Text S1), offers practical insights for predicting product viability under ambient conditions, particularly for export markets [27,28]. The estimated shelf life for thermally sterilized plant-based eggs is 188 days at a storage temperature of 30 °C. Notably, this result suggests a considerably longer shelf life compared to current commercial products in the market, which report a 90–120 day shelf life under refrigeration [12]. This advancement in preservation technology could potentially revolutionize the storage and distribution of plant-based egg alternatives.

#### 3.5.2. Shelf Life Estimation: Gamma Ray Sterilization

**Figure 4:**
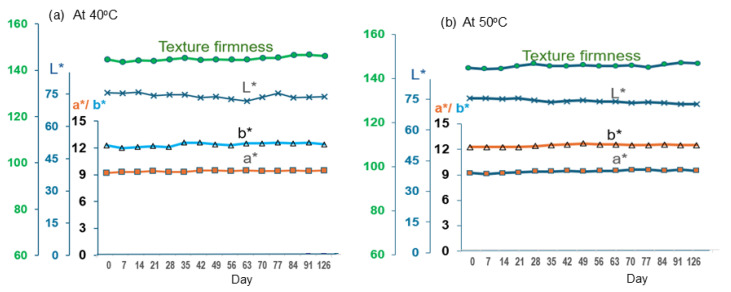
Quality Changes for Gramma ray preserved Plant-Based Hard-Boiled Egg Products: (**a**) at 40 °C and (**b**) at 50 °C. Groups of multiple data points are shown with horizontal braces to indicate significant differences between groups at *p* < 0.05. Data are provided in Supplementary Materials Table S2.

Figure 4a and Figure 4b depict the changes of those properties for the products sterilized using gamma rays for the entire 4 months of accelerated storage at 40 °C and 50 °C, respectively. They demonstrate the superior preservation qualities of the treatment in maintaining quality over thermal pasteurization. Remarkably, microbial stability was maintained longer, with a slight increase observed only by day 117 at 40 °C (Table 9). No microbial changes were evident at 50 °C throughout the testing period, underlining the efficacy of gamma-ray sterilization in extending microbial safety. The increases in TBA levels by day 117 at 40 °C and day 54 at 50 °C, with sensory evaluations confirming the onset of undesirable odors. A shelf life of 253 days at 30 °C for gamma-ray sterilized plant-based eggs far exceeds the 90–120 day refrigeration limits reported for current commercial equivalents. This positions our formulation as a competitive candidate for long-shelf-life plant-based protein products.

The study demonstrates that both pasteurization and gamma-ray sterilization significantly extend the shelf life of plant-based whole hard-boiled eggs, with gamma-ray treatment providing superior results in color preservation, microbial stability, and extended shelf life. These results support the feasibility of employing gamma-ray sterilization for plant-based egg products intended for longer-term storage and suggest its potential for enhancing product safety and quality in global markets. The findings, as illustrated in Table 10, indicate promising applications for these preservation methods in maintaining the quality and extending the marketability of plant-based egg products at various storage temperatures.

Both methods significantly extended the shelf life of plant-based whole hard-boiled eggs compared to real boiled eggs, which typically spoil within 1–2 days without refrigeration. Gamma ray sterilization proved more effective, offering an additional 2.15 months of shelf life at room temperature. This makes gamma ray sterilization particularly promising for developing export products, especially for markets in colder climates where even longer shelf lives could be achieved.

## 4. Conclusions

This study successfully developed a long shelf-life, ready-to-eat (RTE) plant-based hard-boiled egg using locally sourced natural ingredients, aiming to stimulate market growth and create new opportunities for producers. The optimal formulas utilize mung bean protein for low allergenic versions and wheat protein for regular versions, with pasteurization at 75 °C for 15 min effectively eliminating microorganisms while maintaining quality. Gamma ray sterilization at 3.5 kGy also proved effective, though sensory qualities declined at higher doses. Accelerated shelf life testing showed significant texture softening by day 84 at 45 °C and by day 49 at 55 °C for pasteurized eggs, while gamma-ray sterilized eggs exhibited microbial growth at 40 °C by day 117 but remained stable at 50 °C. The Q_10_ value predicted a shelf life of approximately 253 days (8.42 months) at 30 °C. This study demonstrated the feasibility of formulating shelf-stable plant-based egg analogues using mung bean and wheat proteins. All samples achieved acceptable sensory scores, and shelf life modeling suggests gamma-sterilized versions may remain viable for over 8 months at ambient temperatures. This innovation not only offers a nutritious and convenient option for special groups such as the elderly, individuals with egg allergies, diet-conscious consumers, and busy individuals, but also demonstrates the technical feasibility of producing low-allergenic, shelf-stable plant-based egg products using locally sourced ingredients. The development of both regular and low-allergenic formulas offers potential for wider product diversity in the plant-based sector. This study provides a foundation for future research on optimizing plant-based egg analogues for extended shelf life, nutritional adequacy, and consumer acceptance. The demonstrated shelf stability and acceptable sensory performance support potential applications in convenience foods and global distribution.

## Figures and Tables

**Table 1:** Physical properties (firmness and color) of plant-based whole hard-boiled eggs formulated with different plant proteins.

Properties	Rice Protein	Ratio of Rice Protein: Mung Bean Protein				
		Mung Bean Protein 0:100	Pea Protein 50:50	Hemp Protein 70:30	Soy Protein 0:100	Wheat Protein 0:100
**Physical properties**						
Texture firmness (mm)	139.8 ± 1.6 ^bc^	**143 ± 1.5 ^bc^**	144.0 ± 1.0 ^b^	145.0 ± 3.5 ^b^	159.0 ± 2.0 ^a^	**125.4 ± 3.0 ^c^**
L*	73.0 ± 0.5 ^a^	**75.7 ± 0.3 ^a^**	71.6 ± 1.3 ^a^	68.2 ± 1.5 ^b^	82.1 ± 1.5 ^a^	**71.6 ± 1.6 ^a^**
a*	10.8 ± 1.5 ^a^	**9.1 ± 1.5 ^a^**	11.0 ± 0.1 ^a^	8.2 ± 0.9 ^a^	9.1 ± 1.0 ^a^	**11.0 ± 1.4 ^a^**
b*	11.7 ± 1.5 **c**	**11.5 ± 1.5 ^c^**	11.6 ± 0.5 ^c^	22.4 ± 2.2 ^ab^	15.6 ± 1.2 ^b^	**29.2 ± 1.0 ^a^**

Note: Different superscript letters in a row indicate statistically significant differences (*p* < 0.05).

**Table 2:** Sensory quality of plant-based whole hard-boiled eggs formulated with different plant proteins.

Sensory Test	Rice Protein	Ratio of Rice Protein: Mung Bean Protein				
		Mung Bean Protein 0:100	Pea Protein 50:50	Hemp Protein 70:30	Soy Protein 0:100	Wheat Protein 0:100
appearance	7.9 ± 0.6 ^a^	**8.2 ± 0.4 ^a^**	8.1 ± 0.2 ^a^	7.7 ± 0.2 ^a^	8.5 ± 0.2 ^a^	**8.6 ± 0.4 ^a^**
color	8.2 ± 0.3 ^a^	**8.5 ± 0.2 ^a^**	8.2 ± 0.4 ^a^	7.6 ± 0.3 ^a^	8.6 ± 0.4 ^a^	**8.5 ± 0.3 ^a^**
smell	7.7 ± 0.8 ^a^	**6.8 ± 0.4 ^a^**	6.9 ± 0.4 ^a^	7.7 ± 1.2 ^a^	7.4 ± 0.7 ^a^	**7.5 ± 0.5 ^a^**
taste	7.5 ± 0.4 ^a^	**6.9 ± 0.6 ^a^**	7.2 ± 0.3 ^a^	7.2 ± 0.3 ^a^	7.6 ± 0.3 ^a^	**7.5 ± 0.2 ^a^**
texture	7.3 ± 0.4 **^a^**	**6.4 ± 0.5 ^bc^**	7.4 ± 1.2 **^a^**	6.2 ± 0.5 **^c^**	6.7 ± 0.2 **^b^**	**7.0 ± 0.5 ^ab^**
Overall liking	7.4 ± 0.2 ^a^	**7.2 ± 0.3 ^a^**	7.1 ± 1.3 ^a^	6.5 ± 0.3 ^a^	7.0 ± 0.6 ^a^	**7.1 ± 0.3 ^a^**

Note: Different letters within the same row indicate statistically significant differences (*p* < 0.05), as determined by one-way ANOVA followed by Tukey’s HSD test.

**Table 3:** Protein in real eggs and selected plant-based egg formulas.

	Contents						
	Rice Protein	Mung Bean	Pea Protein	Hemp Protein	Soy Protein	Wheat Protein	Limitation
Total protein (g/egg)	7.38 ± 1.34 ^a^	7.08 ± 0.94 ^a^	7.23 ± 1.15 ^a^	6.77 ± 0.93 ^a^	7.35 ± 1.22 ^a^	7.08 ± 1.24 ^a^	6–7 g/egg
Oil content (g/egg)	1.92 ± 0.44 ^a^	1.29 ± 0.35 ^b^	1.98 ± 0.36 ^a^	0.36 ± 0.13 ^d^	0.36 ± 0.11 ^d^	0.72 ± 0.10 ^c^	<1.5 g/egg

Note: Data are presented as mean ± standard deviation. Different superscript letters in the same row indicate significant differences (*p* < 0.05) based on one-way ANOVA followed by Tukey’s HSD test.

**Table 4:** Physical and sensory quality pasteurization on low allergenic hard-boiled eggs made from mung bean protein.

Quality	Before Heating	Temperature (°C)						Criteria
		75		80		85		
Physical		15 min	20 min	15 min	20 min	15 min	20 min	
Firmness(mm)	143.0 ± 1.5	143.0 ± 1.0 ^a^	143.9 ± 1.3 ^a^	144.1 ± 1.1 ^a^	144.6 ± 1.0 ^a^	144.9 ± 1.7 ^a^	145.0 ± 1.8 ^a^	Small different from before heating
Color L*	75.7 ± 0.3	75.7 ± 3.8 ^a^	75.6 ± 3.6 ^a^	74.5 ± 3.7 ^a^	74.7 ± 3.5 ^a^	74.5 ± 5.4 ^a^	74.0 ± 5.6 ^a^	
a*	9.1 ± 1.5	9.2 ± 1.6 ^a^	9.3 ± 1.4 ^a^	9.4 ± 0.9 ^a^	9.5 ± 0.4 ^a^	9.6 ± 0.5 ^a^	9.7 ± 0.8 ^a^	
b*	11.5 ± 1.5	11.5 ± 1.3 ^a^	11.8 ± 1.4 ^a^	12.0 ± 1.8 ^a^	12.8 ± 1.2 ^a^	13.2 ± 1.1 ^a^	13.6 ± 1.3 ^a^	
Sensory								
Appearance		8.2 ± 0.2 ^a^	8.0 ± 0.0 ^a^	8.0 ± 0.0 ^a^	8.0 ± 0.0 ^a^	8.0 ± 0.0 ^a^	8.0 ± 0.0 ^a^	>6 (9 Scale)
Color		8.0 ± 0.0 ^a^	8.0 ± 0.0 ^a^	8.0 ± 0.0 ^a^	8.0 ± 0.0 ^a^	8.0 ± 0.0 ^a^	8.0 ± 0.0 ^a^	>6 (9 Scale)
Smell		7.4 ± 0.5 ^a^	7.3 ± 0.8 ^a^	7.2 ± 0.6 ^a^	7.1 ± 0.7 ^a^	7.2 ± 0.6 ^a^	7.2 ± 0.6 ^a^	>6 (9 Scale)
Taste		7.0 ± 0.5 ^a^	6.9 ± 0.6 ^a^	7.0 ± 0.0 ^a^	6.7 ± 0.6 ^a^	6.9 ± 0.6 ^a^	6.6 ± 0.5 ^a^	>6 (9 Scale)
Texture		7.0 ± 0.0 ^a^	6.9 ± 0.1 ^a^	6.5 ± 0.2 ^b^	6.4 ± 0.2 ^b^	6.1 ± 0.1 ^bc^	6.0 ± 0.0 ^c^	>6 (9 Scale)
Overall liking		7.0 ± 0.5 ^a^	6.9 ± 0.6 ^a^	7.0 ± 0.0 ^a^	6.9 ± 0.5 ^a^	7.0 ± 0.0 ^a^	6.8 ± 0.4 ^a^	>6 (9 Scale)
Microorganism (CFU/g)								
Total aerobic bacteria		0	0	0	0	0	0	Not detected
Yeast & Mold		0	0	0	0	0	0	Not detected
*E. coil*		0	0	0	0	0	0	Not detected
*Clostridium perfringens*		0	0	0	0	0	0	<100 CFU/g
*Staphylococcus aureus*		0	0	0	0	0	0	Not found in 0.1 g
*Salmonella* spp.		0	0	0	0	0	0	Not found in 25 g
*Bacillus cereus*		0	0	0	0	0	0	<100 CFU/g

Note: Data are presented as mean ± standard deviation. Different superscript letters in the same row indicate significant differences (*p* < 0.05) based on one-way ANOVA followed by Tukey’s HSD test.

**Table 5:** Physical and sensory quality of pasteurization for low-allergenic hard-boiled eggs made from wheat protein.

Quality	Temperature (°C)						Criteria
	75		80		85		
Physical	15 min	20 min	15 min	20 min	15 min	20 min	
Firmness(mm)	127.2 ± 2.2 ^d^	133.1 ± 1.1 ^c^	134.1 ± 2.4 ^c^	138.2 ± 5.0 ^bc^	143.1 ± 1.7 ^b^	153.5 ± 1.5 ^a^	125.4 ± 3.0
Color L*	73.5 ± 1.3 ^a^	73.0 ± 1.0 ^a^	72.5 ± 1.4 ^a^	73.2 ± 1.0 ^a^	72.1 ± 2.5 ^a^	72.0 ± 2.0 ^a^	71.6 ± 1.6
a*	10.8 ± 1.7 ^a^	11.0 ± 1.5 ^a^	11.2 ± 1.1 ^a^	11.0 ± 0.4 ^a^	11.9 ± 1.2 ^a^	11.1 ± 1.5 ^a^	11.0 ± 1.4
b*	28.1 ± 1.4 ^a^	27.8 ± 1.2 ^a^	28.8 ± 1.6 ^a^	28.8 ± 1.5 ^a^	29.3 ± 1.4 ^a^	29.2 ± 1.2 ^a^	29.2 ± 1.0
Sensory							
Appearance	8.0 ± 0.0 ^a^	8.2 ± 0.4 ^a^	8.0 ± 0.0 ^a^	8.0 ± 0.0 ^a^	8.0 ± 0.0 ^a^	8.0 ± 0.0 ^a^	>6 (9 Scale)
Color	8.0 ± 0.0 ^a^	8.0 ± 0.0 ^a^	8.0 ± 0.0 ^a^	8.0 ± 0.0 ^a^	8.0 ± 0.0 ^a^	8.0 ± 0.0 ^a^	>6 (9 Scale)
Smell	7.0 ± 0.0 ^a^	7.3 ± 0.8 ^a^	7.2 ± 0.6 ^a^	7.1 ± 0.7 ^a^	7.2 ± 0.6 ^a^	7.2 ± 0.6 ^a^	>6 (9 Scale)
Taste	7.0 ± 0.0 ^a^	6.9 ± 0.6 ^a^	7.0 ± 0.0 ^a^	6.7 ± 0.6 ^a^	6.9 ± 0.6 ^a^	6.6 ± 0.5 ^a^	>6 (9 Scale)
Texture	7.0 ± 0.0 ^ab^	6.7 ± 0.5 ^a^	6.4 ± 0.2 ^ab^	6.1 ± 0.1 ^b^	6.1 ± 0.1 ^b^	6.0 ± 0.0 ^b^	>6 (9 Scale)
Overall liking	7.0 ± 0.0 ^a^	7.0 ± 0.0 ^a^	6.9 ± 0.5 ^a^	6.9 ± 0.5 ^a^	7.0 ± 0.0 ^a^	6.8 ± 0.4 ^a^	>6 (9 Scale)
Microorganism (CFU/g)							
Total aerobic bacteria	0	0	0	0	0	0	Not detected
Yeast & Mold	0	0	0	0	0	0	Not detected
*E. coil*	0	0	0	0	0	0	Not detected
*Clostridium perfringens*	0	0	0	0	0	0	<100 CFU/g
*Staphylococcus aureus*	0	0	0	0	0	0	Not found in 0.1 g
*Salmonella* spp.	0	0	0	0	0	0	Not found in 25 g
*Bacillus cereus*	0	0	0	0	0	0	<100 CFU/g

Note: Data are presented as mean ± standard deviation. Different superscript letters in the same row indicate significant differences (*p* < 0.05) based on one-way ANOVA followed by Tukey’s HSD test.

**Table 6:** Effects of low-dose gamma irradiation on low allergenic mung bean protein hard-boiled.

Quality	Level (KGy)				Criteria
	0	2	3.5	5	
Physical					
Firmness (mm)	143.0 ± 1.5 ^a^	142.0 ± 2.0 ^a^	144.7 ± 1.7 ^a^	145.0 ± 1.5 ^a^	143.0 ± 1.5
Color L*	75.7 ± 3.8 ^a^	75.5 ± 2.5 ^a^	75.0 ± 1.6 ^a^	72.0 ± 2.0 ^a^	75.7 ± 0.3
a*	9.2 ± 1.6 ^a^	9.2 ± 1.8 ^a^	9.2 ± 1.0 ^a^	9.3 ± 1.5 ^a^	9.1 ± 1.5
b*	12.0 ± 2.3 ^a^	12.6 ± 2.1 ^a^	12.3 ± 2.5 ^a^	12.8 ± 2.4 ^a^	11.5 ± 1.5
Sensory					
Appearance	8.0 ± 0.0 ^a^	6.3 ± 0.5 ^b^	6.2 ± 0.4 ^b^	6.4 ± 0.5 ^b^	>6 (9 Scale)
Color	8.0 ± 0.0 ^a^	6.5 ± 0.5 ^b^	6.5 ± 0.5 ^b^	6.4 ± 0.5 ^b^	>6 (9 Scale)
Smell	8.0 ± 0.0 ^a^	6.5 ± 0.5 ^b^	6.5 ± 0.5 ^b^	5.4 ± 0.7 ^c^	>6 (9 Scale)
Taste	8.0 ± 0.5 ^a^	6.9 ± 0.3 ^b^	6.9 ± 0.6 ^b^	5.3 ± 0.5 ^c^	>6 (9 Scale)
Texture	8.0 ± 0.0 ^a^	7.0 ± 0.0 ^b^	6.8 ± 0.4 ^b^	5.2 ± 0.6 ^c^	>6 (9 Scale)
Overall liking	8.5 ± 0.5 ^a^	6.6 ± 0.5 ^b^	6.7 ± 0.4 ^b^	5.3 ± 0.6 ^c^	>6 (9 Scale)
Microorganism (CFU/g)					
Total aerobic bacteria	1.23 × 10^4^ ± 0.36 ^a^	2.8 × 10^2^ ± 0.31 ^b^	0	0	Not detected
Yeast & Mold	106 ± 8.7 ^a^	10 ± 2.6 ^b^	0	0	Not detected
*E. coil*	0	0	0	0	Not detected
*Clostridium perfringens*	4.3 ± 2.5 ^a^	0 ^b^	0	0	<100 CFU/g
*Staphylococcus aureus*	0	0	0	0	Not found in 0.1 g
*Salmonella* spp.	0	0	0	0	Not found in 25 g
*Bacillus cereus*	0	0	0	0	<100 CFU/g

Note: Data are presented as mean ± standard deviation. Different superscript letters in the same row indicate significant differences (*p* < 0.05) based on one-way ANOVA followed by Tukey’s HSD test.

**Table 7:** Effects of low-dose gamma irradiation on wheat protein hard-boiled eggs.

Quality	Level (KGy)				Criteria
	0	2	3.5	5	
Physical					
Firmness (mm)	125.1 ± 1.8 ^a^	126.8 ± 0.8 ^a^	127.1 ± 0.6 ^a^	128.3 ± 1.1 ^a^	125.4 ± 3.0
Color L*	72.5 ± 0.6 ^a^	72.0 ± 0.8 ^a^	71.8 ± 0.5 ^a^	71.5 ± 0.6 ^a^	71.6 ± 1.6
a*	11.8 ± 1.0 ^a^	11.8 ± 0.8 ^a^	12.0 ± 0.7 ^a^	12.3 ± 0.5 ^a^	11.0 ± 1.4
b*	29.6 ± 0.5 ^a^	29.8 ± 0.4 ^a^	30.1 ± 0.7 ^a^	30.0 ± 1.1 ^a^	29.2 ± 1.0
Sensory					
Appearance	8.0 ± 0.0 ^a^	6.5 ± 0.5 ^b^	6.8 ± 0.4 ^b^	6.3 ± 0.4 ^b^	>6 (9 Scale)
Color	8.0 ± 0.0 ^a^	6.5 ± 0.5 ^b^	6.1 ± 07 ^b^	6.4 ± 0.5 ^b^	>6 (9 Scale)
Smell	7.0 ± 0.0 ^a^	6.4 ± 0.5 ^b^	6.0 ± 0.6 ^b^	5.4 ± 0.5 ^c^	>6 (9 Scale)
Taste	7.0 ± 0.0 ^a^	6.3 ± 0.4 ^b^	6.2 ± 0.8 ^b^	5.3 ± 0.5 ^c^	>6 (9 Scale)
Texture	7.0 ± 0.0 ^a^	6.8 ± 0.4 ^b^	6.2 ± 0.4 ^b^	5.2 ± 0.4 ^c^	>6 (9 Scale)
Overall liking	7.0 ± 0.0 ^a^	6.5 ± 0.5 ^b^	6.3 ± 0.4 ^b^	5.3 ± 0.5 ^c^	>6 (9 Scale)
Microorganism (CFU/g)					
Total aerobic bacteria	1.36 × 10^4^ ± 4.0 ^a^	3.3 × 10^2^ ± 0.40 ^b^	0 ^c^	0 ^c^	Not detected
Yeast & Mold	139 ± 6.6 ^a^	11.0 ± 3.1 ^b^	0 ^c^	0 ^c^	Not detected
*E. coil*	0 ^a^	0 ^a^	0 ^a^	0 ^a^	Not detected
*Clostridium perfringens*	6.3 ± 1.5 ^a^	2.3 ± 2.0 ^b^	0 ^b^	0 ^b^	<100 CFU/g
*Staphylococcus aureus*	0 ^a^	0 ^a^	0 ^a^	0 ^a^	Not found in 0.1 g
*Salmonella* spp.	0 ^a^	0 ^a^	0 ^a^	0 ^a^	Not found in 25 g
*Bacillus cereus*	0 ^a^	0 ^a^	0 ^a^	0 ^a^	<100 CFU/g

Note: Data are presented as mean ± standard deviation. Different superscript letters in the same row indicate significant differences (*p* < 0.05) based on one-way ANOVA followed by Tukey’s HSD test.

**Table 8:** Changes in Total Microbial Count and TBA Value in Plant-Based Hard-Boiled Egg Products Sterilized by Pasteurization Combined with Natural Preservatives.

Day	Temperature 45 °C		Temperature 55 °C	
	Total Microbial Count (CFU/g)	TBA (mmol/kg)	Total Microbial Count (CFU/g)	TBA (mmol/kg)
0	0 ^b^	28.15 ± 0.50 ^c^	0 ^a^	28.15 ± 0.50 ^c^
7	0 ^b^	28.10 ± 0.28 ^c^	0 ^a^	29.84 ± 0.50 ^c^
14	0 ^b^	28.17 ± 0.30 ^c^	0 ^a^	30.02 ± 0.50 ^c^
21	0 ^b^	28.42 ± 0.20 ^c^	0 ^a^	30.12 ± 0.36 ^c^
28	0 ^b^	28.86 ± 0.25 ^c^	0 ^a^	32.28 ± 0.20 ^b^
35	0 ^b^	29.00 ± 0.50 ^bc^	0 ^a^	32.46 ± 0.24 ^b^
42	0 ^b^	29.03 ± 0.55 ^bc^	0 ^a^	32.77 ± 0.25 ^b^
49	0 ^b^	29.46 ± 0.20 ^bc^	**0 ^a^**	**35.18 ± 0.45 ^a^**
56	0 ^b^	30.11 ± 0.20 ^bc^	0 ^a^	35.26 ± 0.40 ^a^
63	0 ^b^	30.42 ± 0.25 ^b^	0 ^a^	36.20 ± 0.55 ^a^
70	0 ^b^	30.76 ± 0.10 ^b^	0 ^a^	36.46 ± 0.20 ^a^
77	0 ^b^	30.82 ± 0.15 ^b^	0 ^a^	36.68 ± 0.46 ^a^
84	**0.66 ± 0.58 ^a^**	**32.47 ± 0.10 ^a^**	0 ^a^	36.92 ± 0.50 ^a^
91	1.66 ± 0.58 ^a^	32.90 ± 0.15 ^a^	0 ^a^	37.10 ± 0.26 ^a^

Note: Values are expressed as mean ± standard deviation. Superscript letters (a, b, c, …) within the same column indicate statistically significant differences (*p* < 0.05).

**Table 9:** Changes in Total Microbial Count and TBA Value in Plant-Based Hard-Boiled Egg Products Sterilized by Gamma Irradiation.

Day	Temperature 40 °C		Temperature 50 °C	
	Total Microbial Count (CFU/g)	TBA (mmol/kg)	Total Microbial Count (CFU/g)	TBA (mmol/kg)
0	0 ^b^	35.02 ± 5.50 ᵈ	0 ^a^	35.02 ± 5.50 ^c^
9	0 ^b^	35.04 ± 5.20 ᵈ	0 ^a^	36.10 ± 5.00 ^c^
18	0 ^b^	35.56 ± 5.30 ᵈ	0 ^a^	35.35 ± 5.50^c^
27	0 ^b^	34.82 ± 5.20 ᵈ	0 ^a^	43.66 ± 5.20 ^b^
36	0 ^b^	36.04 ± 5.50 ᵈ	0 ^a^	42.74 ± 5.50 ^b^
45	0 ^b^	38.42 ± 5.50 ^c^ᵈ	0 ^a^	42.65 ± 5.40 ^b^
54	0 ^b^	39.10 ± 5.60 ^c^ᵈ	**0 ^a^**	**64.06 ± 5.50 ^a^**
63	0 ^b^	41.54 ± 5.20 ^c^ᵈ	0 ^a^	64.52 ± 5.30 ^a^
72	0 ^b^	45.27 ± 5.60 ^c^	0 ^a^	65.85 ± 5.20 ^a^
81	0 ^b^	46.45 ± 5.30 ^c^	0 ^a^	65.30 ± 5.60 ^a^
90	0 ^b^	46.05 ± 5.10 ^c^	0 ^a^	65.20 ± 5.20 ^a^
99	0 ^b^	53.62 ± 5.00 ^bc^	0 ^a^	66.65 ± 5.60 ^a^
108	0 ^b^	53.07 ± 5.20 ^bc^	0 ^a^	66.32 ± 5.50 ^a^
117	**6.6 ± 2.0 ^a^**	**67.12 ± 5.00 ^b^**	0 ^a^	66.50 ± 5.50 ^a^
126	21.0 ± 3.6 ᵃ	78.06 ± 5.50 ᵃ	0 ᵃ	66.65 ± 5.00 ᵃ

Note: Values are expressed as mean ± standard deviation. Superscript letters (a, b, c,...) within the same column indicate statistically significant differences (*p* < 0.05).

**Table 10:** Prediction of shelf life of plant-based whole hard-boiled eggs.

Store temperature °C	Prediction Shelf Life (days)	
	Thermal Pasteurization	Gamma Ray Sterilization
5	715	
10	547	
15	419	
20	320	545
25	245	371
30	188	253

Note: The calculation method is detailed in Supplementary Text S1 (Thermal Pasteurization) and Supplementary Text S2 (Gamma Ray Sterilization).

## Data Availability

The original contributions presented in the study are included in the article/Supplementary Materials, further inquiries can be directed to the corresponding authors.

## Supplementary Materials

The following supporting information can be downloaded at: https://www.mdpi.com/article/10.3390/foods14132220/s1, Table S1: Quality Changes for Pasteurized Plant-Based Hard-Boiled Egg Products Combined with Natural Preservatives; Text S1: Calculation steps for prediction of shelf life for thermal pasteurization; Table S2: Quality Changes for Gramma ray preserved Plant-Based Hard-Boiled Egg Products Combined with Natural Preservatives; Text S2: Calculation steps for prediction of shelf life for gamma irradiation.
