# Supplementary material for: Long Shelf-Life Ready-to-Eat Plant-Based Whole Hard-Boiled Eggs: Low Allergenic and Regular Formulas
Source: Foods. 2025 Jun 24;14(13):2220. doi: 10.3390/foods14132220 (PMC12248480; doi:10.3390/foods14132220)
Supplement: Supplementary file 1 [file foods-14-02220-s001.zip › foods-3642588-supplementary.pdf]

Supplementary Table S1. Quality Changes for Pasteurized Plant-Based Hard-Boiled Egg Products Combined with Natural Preservatives

Day	45ºC						55ºC					
	L*	a*	b*	Texture Firmness	Total microorganism (CFU/g)	TBA (mmole/kg)	L*	a*	b*	Texture Firmness	Total microorganism (CFU/g)	TBA (mmole/kg)
0	75.7±4.4a	9.2±1.6a	11.5±1.3a	143.0±1.1b	0b	28.15±0.50c	75.7±4.4a	9.2±1.6a	11.5±1.3a	143.0±1.1c	0a	28.15±0.50c
7	75.1±3.2a	9.3±1.4a	11.6±1.1a	143.2±2.1b	0b	28.10±0.28c	74.8±4.5a	9.2±1.4a	11.5±1.5a	143.4±2.4c	0a	29.84±0.50c
14	75.5±3.5a	9.3±1.5a	11.7±1.5a	143.4±2.5b	0b	28.17±0.30c	73.0±4.0a	9.1±1.4a	11.7±1.5a	143.6±2.1c	0a	30.02±0.50c
21	75.7±3.7a	9.4±1.4a	11.7±2.0a	143.3±2.3b	0b	28.42±0.20c	68.7±4.6a	9.2±1.6a	11.8±1.6a	144.8±2.5c	0a	30.12±0.36c
28	74.2±2.5a	9.3±1.8a	11.8±2.2a	144.2±1.1b	0b	28.86±0.25c	67.6±4.2a	9.1±1.7a	12.0±1.6a	144.7±1.5c	0a	32.28±0.20b
35	74.4±3.1a	9.2±1.9a	11.8±2.3a	143.7±2.3b	0b	29.00±0.50bc	67.0±4.5a	9.3±1.8a	11.6±1.5a	145.6±2.6c	0a	32.46±0.24b
42	73.0±3.0a	9.1±1.6a	12.0±2.5a	144.4±1.7b	0b	29.03±0.55bc	66.7±4.6a	9.4±1.3a	12.3±1.2a	146.7±2.0c	0a	32.77±0.25b
49	73.2±3.7a	9.2±1.5a	11.9±2.2a	144.5±1.6b	0b	29.46±0.20bc	64.3±4.4b	9.5±1.4a	12.4±1.1a	156.5±1.1b	0a	35.18±0.45a
56	72.5±3.0a	9.4±1.6a	12.0±1.5a	144.2±1.3b	0b	30.11±0.20bc	60.9±3.0bc	9.6±1.0a	12.5±1.1a	156.6±1.0b	0a	35.26±0.40a
63	71.5±3.7a	9.5±1.5a	12.0±1.2a	145.0±1.1b	0b	30.42±0.25b	58.7±3.3bc	9.7±1.7a	12.7±1.3a	157.8±1.3b	0a	36.20±0.55a
70	71.6±2.4a	9.5±1.4a	12.3±1.7a	145.1±1.7b	0b	30.76±0.10b	57.0±3.0bc	9.6±1.0a	12.5±1.1a	161.7±1.5a	0a	36.46±0.20a
77	70.4±2.3a	9.4±1.3a	12.4±1.5a	145.2±1.7b	0b	30.82±0.15b	56.1±3.2c	9.6±1.0a	12.5±1.1a	162.0±1.5a	0a	36.68±0.46a
84	64.6±2.5b	9.5±1.5a	12.4±1.0a	151.4±1.7a	0.66±0.58a	32.47±0.10a	55.2±3.4c	9.7±1.7a	12.7±1.5a	164.4±1.4a	0a	36.92±0.50a
91	63.2±2.2b	9.5±1.8a	12.4±1.5a	151.5±1.4a	1.66±0.58a	32.90±0.15a	51.5±3.5c	9.7±1.7a	12.7±1.0a	165.1±1.5a	0a	37.10±0.26a

Note: Data are presented as mean ± standard deviation. Different letters in the same column indicate significant differences ($p < 0.05$)

Supplementary S2: Calculation steps for prediction of shelf life for thermal pasteurization

The calculation steps for prediction of shelf life for thermal pasteurization From the study results, significant changes in L* color value, texture firmness, TBA value, and microbial count, as well as sensory evaluations of texture and overall preference in samples stored at 45°C, allowed the end of shelf life for the plant-based boiled egg product to be determined as 84 days. Similarly, significant changes in L* color value, texture firmness, and TBA value, along with sensory evaluations of color, texture, and overall preference at 55°C, indicated an end-of-shelf-life of 49 days for the plant-based boiled egg product. By using these analytical results to predict shelf life, it is possible to calculate the Q10 value for the product and determine the shelf life at 30°C, which represents the average annual temperature or room temperature in Thailand, as per the following equation.

Q10	=	$\theta S(T) / \theta S(T+10)$
	=	$\theta S(45) / \theta S(55)$
	=	84 / 49
	=	1.71
From Q1	=	$\theta 10^{0.1}$
	=	$1.71^{0.1} = 1.0551$
$Q1^{45-30}$	=	1.0551^{15}
	=	$\theta S(30) / \theta S(45)$
	=	$\theta S(30) / 84$
$\theta S(30)$	=	$1.0551^{15} \times 84$
	=	188 วัน

From the shelf-life calculation at 30°C, it was found that the product can be stored for 188 days, or approximately 6 months and 8 days. Typically, real boiled eggs spoil quickly and cannot be kept unrefrigerated for more than 1-2 days. Therefore, this developed plant-based boiled egg product can be stored at room temperature for half a year. Additionally, with a Q10 value of 1.71, it is possible to calculate the Q1 value to further predict shelf life at other temperatures. For example, if stored in a refrigerator at 5-10°C, it can be stored for approximately 1 year, as shown in **Table 10 (In the article)**

Supplementary Table S3 Quality Changes for Gramma ray preserved Plant-Based Hard-Boiled Egg Products Combined with Natural Preservatives

Day	40ºC						50ºC					
	L*	a*	b*	Texture Firmness	Total microorganism (CFU/g)	TBA (mmole/kg)	L*	a*	b*	Texture Firmness	Total microorganism (CFU/g)	TBA (mmole/kg)
0	75.7±3.8a	9.2±1.6a	12.3±2.0a	144.7±2.0a	0b	35.02±5.5d	75.7±3.8a	9.2±1.6a	12.3±2.0a	144.7±2.0a	0a	35.02±5.50c
9	75.4 ±3.5a	9.3±1.5a	12.0±1.6a	143.5 ±2.0b	0b	35.04±5.2d	75.6±3.4a	9.1±1.8a	12.3±1.2a	144.3 ±1.8a	0a	36.10 ±5.0c
18	75.8 ±3.0a	9.3±1.2a	12.1±1.7a	144.2 ±2.0b	0b	35.56±5.3d	75.2 ±3.7a	9.2±1.6a	12.3±1.2a	144.5 ±1.5a	0a	35.35 ±5.5c
27	74.2 ±2.7a	9.4±1.4a	12.2±2.2a	144.0 ±1.6a	0b	34.82±5.2d	75.7±3.4a	9.3±1.5a	12.3±1.6a	145.6 ±1.5a	0a	43.66 ±5.2b
36	74.7 ±3.0a	9.3±1.5a	12.1±1.9a	144.7 ±1.5a	0b	36.04 ±5.5d	74.6±3.2a	9.4±1.4a	12.4±1.5a	146.6 ±1.5a	0a	42.74 ±5.5b
45	74.6 ±3.5a	9.3±1.5a	12.6±2.5a	145.3 ±1.6a	0b	38.42 ±5.5cd	73.7±3.5a	9.4±1.7a	12.5±1.5a	145.6 ±1.7a	0a	42.65 ±5.4b
54	73.3±3.8a	9.5±1.4a	12.6±2.0a	144.4 ±1.8a	0b	39.10±5.6cd	74.1 ±3.2a	9.5±1.4a	12.6±1.4a	145.7 ±1.8a	0a	64.06 ±5.5a
63	73.7 ±3.9a	9.5±1.7a	12.4±2.5a	144.7 ±1.8a	0b	41.54±5.2cd	74.6 ±3.5a	9.4±1.7a	12.7±1.4a	146.1 ±1.5a	0a	64.52 ±5.3a
72	72.7±3.2a	9.4±1.2a	12.3±1.6a	144.5 ±1.5a	0b	45.27±5.6c	74.0±2.5a	9.5±1.7a	12.6±1.7a	145.6 ±1.2a	0a	65.85 ±5.2a
81	71.8 ±3.5a	9.5±1.5a	12.5±1.5a	144.5 ±1.5a	0b	46.45±5.3c	73.8±3.8a	9.5±1.4a	12.6±1.5a	145.7 ±1.8a	0a	65.30 ±5.6a
90	73.6 ±3.2a	9.4±1.5a	12.5±1.5a	145.3 ±1.6a	0b	46.05±5.1c	73.3±3.5a	9.6±1.5a	12.5±1.1a	146.5 ±1.6a	0a	65.20 ±5.2a
99	75.3 ±3.7a	9.4±1.6a	12.6±2.0a	145.4 ±1.5a	0b	53.62±5.0bc	73.6 ±3.3a	9.6±1.3a	12.5±1.1a	145.2 ±1.6a	0a	66.65 ±5.6a
108	73.4±3.5a	9.5±1.1a	12.5±2.2a	146.6±1.5a	0b	53.07±5.2bc	73.4±3.1a	9.5±1.5a	12.6±1.5a	146.4 ±2.0a	0a	66.32 ±5.5a
117	73.5 ±3.4a	9.4±1.2a	12.6±2.5a	146.7±1.8a	6.6±2.0a	67.12±5.0b	72.7±3.5a	9.6±1.6a	12.5±1.4a	147.1 ±1.8a	0a	66.50 ±5.5a
126	73.7 ±3.2a	9.5±1.4a	12.4±2.5a	146.1 ±1.5a	21.0±3.6a	78.06±5.5a	72.7±3.2a	9.5±1.7a	12.5±1.5a	146.8±1.8a	0a	66.65 ±5.0a

Note: Data are presented as mean ± standard deviation. Different letters in the same column indicate significant differences ($p < 0.05$)

Supplementary S4: Calculation steps for prediction of shelf life for gamma irradiation

The study results, showing a significant change in rancidity value (TBA) and sensory odor in samples stored at 40°C, allow the end of shelf life for plant-based boiled egg products to be determined as 117 days. Similarly, a significant change in TBA values in samples stored at 50°C indicates an end-of-shelf-life of 54 days for the plant-based boiled egg products. By using these analytical results to predict shelf life, it is possible to calculate the Q10 value for the product, as well as the shelf life at 30°C, which represents the average annual temperature or room temperature in Thailand, as per the following equation.

Q10	=	ɵS(T) / ɵS(T+10)
	=	ɵS(40) / ɵS(50)
	=	117 / 54
	=	2.17
From Q1	=	$ɵ10^{0.1}$
	=	$2.17^{0.1}$ = 1.08
$Q1^{40-30}$	=	1.08^{10}
	=	ɵS(30) / ɵS(45)
	=	ɵS(30) / 117
ɵS(30)	=	1.08^{10} x 117
	=	252.72 days

From the shelf-life calculation at 30°C, it was found that the product can be stored for 253 days, or approximately 8.42 months. Typically, real boiled eggs spoil quickly and cannot be kept unrefrigerated for more than 1-2 days. Therefore, this irradiated plant-based boiled egg product developed can be stored at room temperature for up to 8 months. Additionally, with a Q10 value of 2.17, it is possible to calculate the Q1 value to further predict shelf life at other temperatures.

The data obtained is interesting in that the shelf-life extension technology for plant-based boiled eggs using irradiation appears suitable for further development as an export product to cold-climate countries that recognize irradiated food products. This product can be stored at a temperature of 20-25°C for an extended period of 1-1.5 years, as shown in **Table 10 (In the article)**
